# Supplementary material for: Elaboration of massage technique for semen collection and examination of semen characteristics in chinchilla (Chinchilla lanigera)
Source: PLoS One. 2023 Aug 31;18(8):e0290441. doi: 10.1371/journal.pone.0290441 (PMC10471009; doi:10.1371/journal.pone.0290441)
Supplement: Attachment — Submitted filename: Response to Reviewers.docx [file pone.0290441.s003.docx]

Dear Reviewer,

thank you for your work, the change is you have requested, have been made.

**1. Inconsistent reference list
- There is inconsistent in writing the reference list. Author(s) should check the journal's format**

We check the format references, and we have corrected the incorrect information and the inconsistencies.

**2. The Fig 1 & Fig 2 should be labelled
-A point of interest in the Fig 1 & Fig 2 should be precisely labelled**

Based on your request we proposed the following changes to the title of the figures:

Fig 1. Collection of sperm sample on a gauze pad whit massage technique base on digital manipulation

Fig 2. Differentiating between live (white) and dead (pink) chinchilla spermatozoa in a stained smear using aniline blue and eosin
